# Chromosomal Localization of Genes Conferring Desirable Agronomic Traits from Wheat-*Agropyron cristatum* Disomic Addition Line 5113

**DOI:** 10.1371/journal.pone.0165957

**Published:** 2016-11-08

**Authors:** Qingfeng Li, Yuqing Lu, Cuili Pan, Miaomiao Yao, Jinpeng Zhang, Xinming Yang, Weihua Liu, Xiuquan Li, Yajun Xi, Lihui Li

**Affiliations:** 1 College of Agronomy, Northwest A&F University, Yangling, Shaanxi, 712100, China; 2 National Key Facility for Crop Gene Resources and Genetic Improvement, Institute of Crop Science, Chinese Academy of Agricultural Sciences, Beijing, 100081, China; Huazhong University of Science and Technology, CHINA

## Abstract

Creation of wheat-alien disomic addition lines and localization of desirable genes on alien chromosomes are important for utilization of these genes in genetic improvement of common wheat. In this study, wheat-*Agropyron cristatum* derivative line 5113 was characterized by genomic *in situ* hybridization (GISH) and specific-locus amplified fragment sequencing (SLAF-seq), and was demonstrated to be a novel wheat-*A*. *cristatum* disomic 6P addition line. Compared with its parent Fukuhokomugi (Fukuho), 5113 displayed multiple elite agronomic traits, including higher uppermost internode/plant height ratio, larger flag leaf, longer spike length, elevated grain number per spike and spikelet number per spike, more kernel number in the middle spikelet, more fertile tiller number per plant, and enhanced resistance to powdery mildew and leaf rust. Genes conferring these elite traits were localized on the *A*. *cristatum* 6P chromosome by using SLAF-seq markers and biparental populations (F_1_, BC_1_F_1_ and BC_1_F_2_ populations) produced from the crosses between Fukuho and 5113. Taken together, chromosomal localization of these desirable genes will facilitate transferring of high-yield and high-resistance genes from *A*. *cristatum* into common wheat, and serve as the foundation for the utilization of 5113 in wheat breeding.

## Introduction

Common wheat (*Triticum aestivum*, genome AABBDD) has a large number of wild relatives, which are considered as valuable gene resources for wheat genetic improvement [1]. Wheat-alien disomic addition lines are usually considered as key materials to transfer alien superior genes into common wheat, which play important roles in numerous applications, such as investigating genetic effects of alien chromosomes under wheat background, determining chromosomal localization of some desirable genes, purifying alien chromosomes or chromosome arms via flow sorting followed by construction of BAC libraries [2,3]. Therefore, creation of wheat-alien disomic addition lines and chromosomal localization of their elite genes are important for wheat genetic improvement. Thus far, plenty of wheat-alien addition lines derived from multiple species of *Triticeae* have been obtained. These *Triticeae* species include *Hordeum vulgare* L. [4–7], *Secale cereale* L. [8,9], *Aegilops speltoides* [10,11] and *Haynaldia villosa* [12,13] and so on. However, most wheat-alien disomic addition lines could not be directly used in wheat breeding due to the presence of linkage drag. Therefore, they are usually induced to produce various translocation or introgression lines, such as wheat-*Psathyrostachys huashanica* translocation line H9020-17-15 [14], wheat *Haynaldia villosa* translocation line 6VS/6AL [15], wheat-rye translocation line 1BL/1RS [16], and wheat-*Thinopyrum pointicum* introgression lines II-1-8 and II-2 [17]. Before addition lines are induced to produce translocation or introgression lines, localization of genes conferring elite agronomic traits were usually considered as a fundamental prerequisite.

Morphological, biochemical and molecular markers are indispensable in localizing alien genes on chromosomes. Compared with other markers, PCR-based molecular markers are much more popular due to their high efficiency and throughput. Specific-locus amplified fragment sequencing (SLAF-seq) marker is one of the PCR-based markers, which is developed from the SLAF-seq technology [18]. SLAF-seq markers have been widely used in chromosomal localization of alien genes [19,20], determination of homoeologous relationships for alien chromosomes [21,22], and genetic map constructions [23–25]. For example, 89 SLAF-seq markers specific to *Th*. *elongatum* 7E chromosome were successfully developed by the SLAF-seq technology [21]; the genetic maps of common carp (*Cyprinus carpio* L.), sesame (*Sesamum indicum* L.) and soybean (*Glycine max* (L.) Merr.) were constructed by the SLAF-seq technology [23–25]. In addition, a high-density genetic map of *Agropyron* Gaertn. was constructed by the SLAF-seq technology in our previous study [26].

*Agropyron cristatum* (L.) Gaertn. (2n = 4x = 28; genome PPPP) is a wild relative of wheat, and displays several superior traits, such as higher grain numbers, multiple fertile tiller numbers, and elevated resistance to biotic and abiotic stresses [27,28]. Pioneering work on the utilization of *A*. *cristatum* have been carried out nearly three decades ago, and a series of wheat-*A*. *cristatum* derivative lines have been obtained from the cross between common wheat cv. Fukuhokomugi (Fukuho, 2n = 6x = 42; AABBDD) and *A*. *cristatum* [29,30], including addition, translocation and introgression lines [31–35]. 5113 was one of the wheat-*A*. *cristatum* disomic addition lines, and the two added alien *A*. *cristatum* chromosomes were homoeologous to group 6 of common wheat. 5113 displays multiple elite agronomic traits such as higher yield and disease resistance. However, whether these elite traits were conferred by desirable genes located on 6P chromosomes has not been determined. Therefore, these desirable genes were localized using SLAF-seq markers and genetic populations in this study.

## Materials and Methods

### Materials

5113 was originally produced from the cross between *A*. *cristatum* accession Z559 and Fukuho, followed by self-pollination for six generations [31]. F_1_, BC_1_F_1_ and BC_1_F_2_ populations were then constructed from the crosses between 5113 and Fukuho, using Fukuho as the recurrent parent. Different types of wheat-*A*. *cristatum* disomic addition lines, each containing only one pair of P chromosomes, were used as controls to determine the homoelogy relationship of 5113 with wheat chromosomes. These wheat-*A*. *criatatum* disomic addition lines were as follows: II-3-1a (containing one pair of 1P chromosomes), II-9-3 (containing one pair of 2P chromosomes), II-11-1a (containing one pair of 3P chromosomes), II-21-2 (containing one pair of 4P chromosomes), II-11-1b (containing one pair of 5P chromosomes), II-5-1 (containing one pair of 7P chromosomes). Besides, Wheat-*A*. *cristatum* disomic 6P addition line 4844–12 was specially chosen as the control to compare the differences of 6P chromosomes. All the materials were provided by the Crop Germplasm Resources Research Center of the Institute of Crop Science, Chinese Academy of Agricultural Sciences (CAAS), Beijing, China.

### Methods

#### GISH analysis and meiosis observation

Genomic *in situ* hybridization (GISH) was used to analyze the chromosomal composition of 5113 and the progenies of the genetic populations as previously described [33]. Genomic DNA was isolated using the modified CTAB method [36]. *Agropyron cristatum* DNA (labeled with Dig-Nick-Translation Mix) (Roche, Mannheim, Germany) and Fukuho genomic DNA was used as the probe and blocker, respectively. For meiosis studies, the procedures were described by Jauhar and Peterson [37]. Briefly, pollen mother cells (PMCs) at metaphase I (MI) stage were fixed in Carnoy’s solution (ethanol:chloroform:acetic acid, 6:3:1, by volume) for 24 h. Wheat and *A*. *cristatum* chromosomes were pseudo-colored as blue and red, respectively. All cytological images were taken under an OLYMPUS AX80 (Olympus Corporation, Tokyo, Japan) fluorescence microscope and captured with a CCD camera (Diagnostic Instruments, Sterling Heights, MI, USA).

#### SLAF library construction and data analysis

The SLAF library of 5113 was constructed as previously described by Sun et al. [23]. Briefly, 2 ug genomic DNA of 5113 was digested with *Nla*III/*Mse*I (New England Biolabs, Beverly, MA, USA). Fragments between 200 and 300 bp (not including adapter sequence indexes and adaptors) were isolated, and then subjected to PCR for paired-end sequencing (40bp each end) by Illumina HiSeq 2500 (Illumina, San Diego, CA, USA) at Biomarker Technologies Corporation in Beijing, China. Analysis of these SLAF-seq data were conducted as follows: Firstly, reads were filtered based on their quality, leaving high-quality clean reads; high-quality reads with high sequence depth (≧ 3) and similarity (≧ 95%) were clustered into single SLAF groups by using BLAT software (-tileSize = 10, -stepSize = 5); Secondly, six reads at most were selected as the representatives of each SLAF group (referred to as SLAF-tagI). Thirdly, all the SLAF-tagIs were blasted with wheat genomes, and those with higher identity (> 90%) were considered from wheat genome and then filtered out; only those with lower identity (< 90%) were referred to as SLAF-tagII. Fourthly, all the SLAF-tagIIs were blasted with the SLAF markers located on the genetic map of *Agropyron* Gaertn. published before [26], and only those with higher identity (> 90%) were considered as the SLAF-tags specific to *A*. *cristatum* 6P chromosome in 5113.

#### Development of SLAF-seq markers

In order to trace *A*. *cristatum* 6P chromosomal segments effectively, SLAF-seq markers were developed based on the sequences of SLAF-tags specific to *A*. *cristatum* 6P chromosome in 5113, PCR was conducted as previously described by Lu et al. [34], and the PCR products were separated on 0.8% agarose gel.

#### Evaluation of agronomic traits

All the materials were planted in 2.0 m rows, spaced 30 cm apart at Xinxiang experiment station in Henan province, China. A number of agronomic traits were evaluated, including plant height, uppermost internode, flag leaf size, grain number per spike, spikelets per spike, thousand-grain weight, and disease responses. A mixture of currently prevalent isolates of *Blumeria graminis f*. *sp*. *tritici* was used to evaluate the powdery mildew resistance of wheat plants at the adult stage, and disease responses were scored as described by Sheng et al. [38]. For leaf rust resistance, mixed *P*. *triticina* pathotypes isolates were used [39,40], and disease responses were recorded with 0–4 raking, where 0–2 were considered resistant and 3–4 susceptible [41]. Statistical analysis system software version 9.2 was used for statistical analyses (SAS Institute, Cary, NC, USA).

## Results

### Cytological characterization of 5113

**Fig 1:**
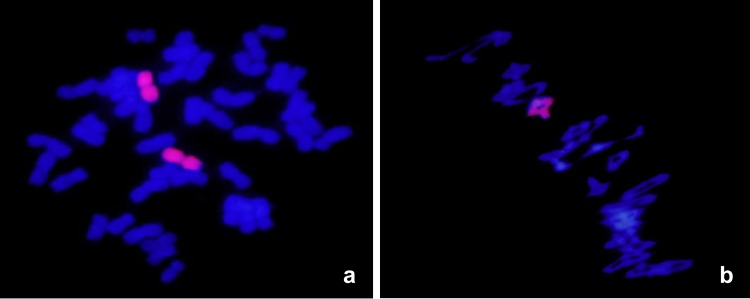
GISH analysis of the root cells and pollen mother cells of 5113. (a), GISH analysis showing that 5113 contained 44 chromosomes including 42 wheat chromosomes (shown in blue) and two *A*. *cristatum* chromosomes (shown in red); (b), 5113 possessed 22 bivalents including 21 wheat bivalents (shown in blue) and one *A*. *cristatum* bivalent (shown in red) at metaphase I (MI) stage of the pollen mother cells (PMCs).

To cytologically characterize the genetic constitution of 5113, root cells and pollen mother cells (PMCs) were used (Fig 1 and Table 1). There were 44 chromosomes in the root cells of 5113, including 42 wheat chromosomes (shown in blue) and two *A*. *cristatum* chromosomes (shown in red) (Fig 1A). Chromosomal configuration at the metaphase I of 120 PMCs were investigated in 5113, showing 2n = 22 II, with averages of 0.6 univalents, 2.29 rod bivalents, and 19.41 ring bivalents (Table 1). Twenty-one wheat bivalents (shown in blue) and one *A*. *cristatum* bivalent (shown in red) were shown in Fig 1B, showing regular chromosome pairing behaviors. Taken together, all these results indicated that 5113 was a stable wheat-*A*. *cristatum* disomic addition line.

**Table 1:** Chromosome pairing during metaphase I in the pollen mother cells of 5113 and Fukuho.

Materials	No. of chromosomes	No. of observed cells	Chromosome configuration			
			Univalent	Bivalent		
				Rod	Ring	Total
**5113**	**44**	**120**	**0.6**	**2.29**	**19.41**	**21.7**
			**(0–2)**	**(0–3)**	**(19–22)**	**(21–22)**
**Fukuho**	**42**	**120**	**0.06**	**1.95**	**19.32**	**20.97**
			**(0–2)**	**(1–4)**	**(17–21)**	**(20–21)**

### Development of SLAF-seq markers specific to 6P chromosome using the SLAF-seq technology

**Fig 2:**
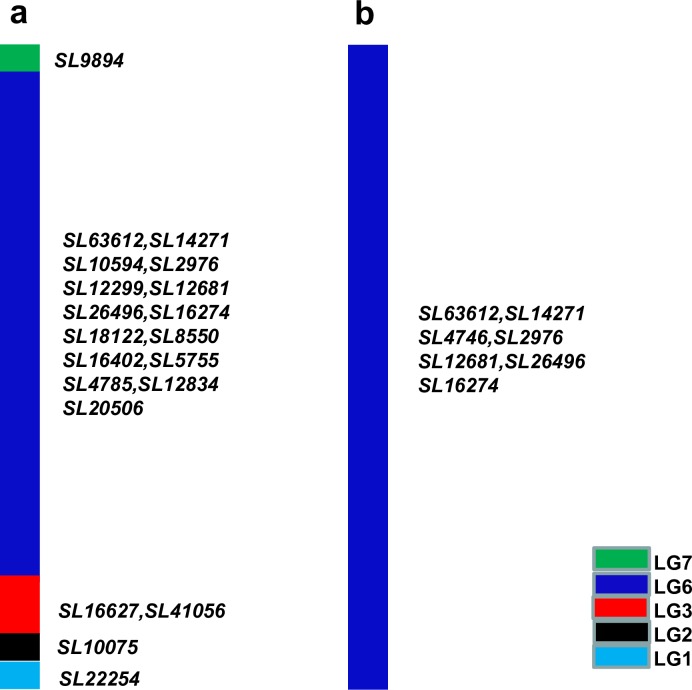
SLAF-tags acquired from 5113 (a) and 4844–12 (b).

In order to acquire P chromosome-specific markers, the SLAF-seq technology was used on 5113. Meanwhile, 4844–12 was chosen as the control. 11,367,661 clean reads (clustered into 50,468 SLAF groups) and 747,660 clean reads (clustered into 27,379 SLAF groups) were acquired from 5113 and 4844–12, respectively. Six reads at most were selected from each SLAF group as representatives, and then 28,948 and 11,454 reads were acquired from 5113 and 4844–12, respectively (thereafter referred to as SLAF-tagI). All the SLAF-tagI sequences were used as queries to search against the wheat genome, and 4,295 and 1,091 tags with lower identity (< 90%) to their hits were referred to as SLAF-tagIIs. When SLAF-tagII sequences were used as queries to search SLAF markers located on the genetic map of *Agropyron* Gaertn., 20 and seven SLAF-tagIIs with high identity (≧ 90%) were obtained from 5113 and 4844–12, respectively (Fig 2). All the seven SLAF-tags from 4844–12 were located on the linkage group (LG) 6 of *Agropyron* Gaertn., indicating that 4844–12 was a 6P disomic addition line. The result was consistent with before [31]. 15 out of 20 SLAF-tags (accounting for 75%) from 5113 were localized on LG6, and others were localized on other different LGs (Fig 2A), such as one tag on LG1, one tag on LG2, two tags on LG3, and one tag on LG7. These results suggested that 5113 was also a 6P disomic addition line, but it contained 6P chromosomes different from those of 4844–12.

### Evaluation of the agronomic traits of 5113, Fukuho, and 4844–12

**Fig 3:**
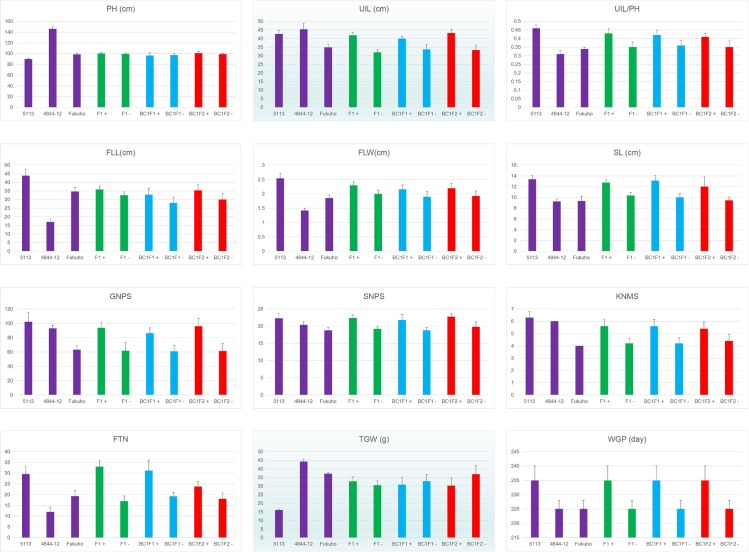
Histograms showing the agronomic traits of 5113, Fukuho, 4844–12 and genetic populations produced from the crosses between 5113 and Fukuho. PH, Plant height; UIL, Uppermost internode length; FLL, Flag leaf length; FLW, Flag leaf width; SL, Spike length; GNPS, Grain number per spike; SNPS, Spikelet number per spike; KNMS, Kernel number in the middle spikelet; TGW, Thousand-grain weight; WGP, Whole growth period; FTN, Fertile tiller number per plant.

**Fig 4:**
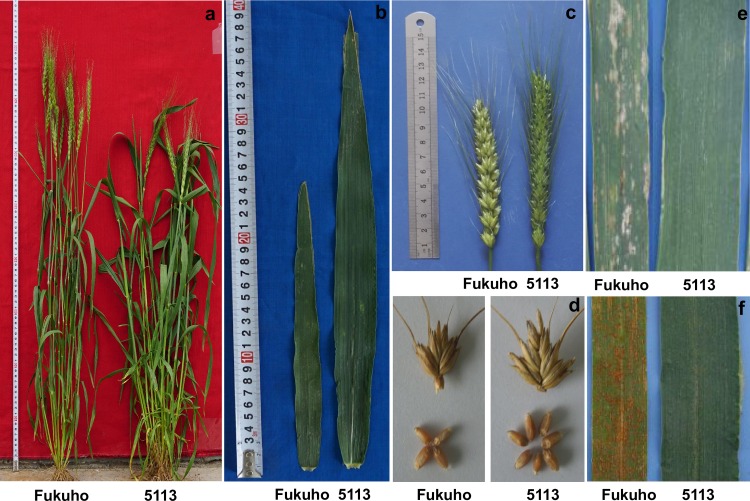
Morphological traits of 5113 and Fukuho. whole plants (a), flag leaves (b), spikes (c), spikelets with grains (d), disease responses to powdery mildew (e) and leaf rust (f).

A number of important agronomic traits were investigated in 5113, common wheat cv. Fukuho and wheat-*A*. *cristatum* 6P addition line 4844–12. As shown in Fig 3 and S1 Table, 5113 showed obviously different agronomic traits from those of Fukuho and 4844–12. Compared with Fukuho, 5113 showed higher ratio between uppermost internode and plant height, larger flag leaf, longer spike length, more fertile tiller number per plant, more spikelet number per spike, more grains per spike and more kernels in the middle spikelet. Besides, 5113 displayed enhanced resistance to powdery mildew and leaf rust, lower thousand-grain weight, and longer whole growth period compared with Fukuho. Compared with the wheat-*A*. *cristatum* 6P addition line 4844–12, 5113 still exhibited higher ratio between uppermost internode and plant height, larger flag leaf, longer spike length, more spikelet number per spike, more fertile tiller number, lower thousand-grain weight and longer whole growth period. The morphologies of whole plants, flag leaves, spikes, spikelets with grains from 5113 and Fukuho were shown in Fig 4A–4D. The responses to powdery mildew and leaf rust were presented in Fig 4E and 4F. All these results suggested that 5113 was a novel wheat-*A*. *cristatum* disomic 6P addition line different from 4844–12, which possessed multiple desirable traits that could be used for wheat genetic improvement.

### *A*. *cristatum* 6P chromosomal segments could be traced by SLAF-seq markers

**Fig 5:**
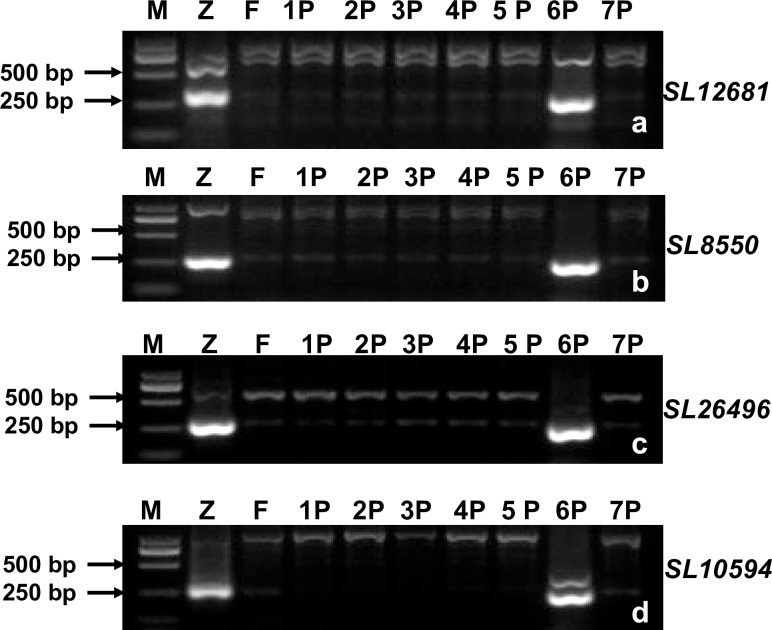
Development of SLAF-seq markers specific for *A*. *cristatum* 6P chromosome. (a-d), PCR patterns of four SLAF-seq markers *SL12681* (a), *SL8550* (b), *SL26496* (c) and *SL10594* (d) M, Marker; Z, *A*. *cristatum* accession Z559; F, Fukuho; 1P-7P, wheat-*A*. *cristatum* disomic addition lines, each of which contained only one pair of P chromosomes (from 1P to 7P).

**Fig 6:**
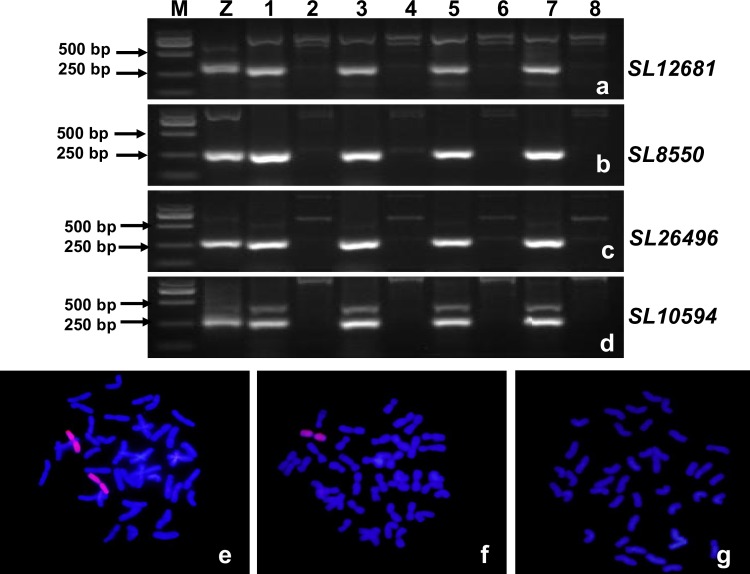
Genotyping of the progenies of the genetic populations using SLAF-seq markers and GISH. (a-d), PCR patterns of four SLAF-seq markers *SL12681* (a), *SL8550* (b), *SL26496* (c) and *SL10594* (d) M, Marker; Z, *A*. *cristutum* accession Z559; 1, 5113; 2, Fukuho; 3, 5113 × Fukuho F_1_ plant with 6P chromosome; 4, 5113 × Fukuho F_1_ plant without 6P chromosome; 5, One progeny from the 5113 × Fukuho BC_1_F_1_ population with 6P chromosome; 6, One progeny from the 5113 × Fukuho BC_1_F_1_ population without 6P chromosome; 7, One progeny from the 5113 × Fukuho BC_1_F_2_ population with 6P chromosome; 8, One progeny from the 5113 × Fukuho BC_1_F_2_ population without 6P chromosome; e-g, GISH analysis of the progenies with three genotypes containing 44 (e), 43(f) and 42 (g) chromosomes, respectively.

Based on the sequences of 15 SLAF-tags on LG6, 15 SLAF-seq markers were developed. The primer sequences of these markers were listed in S2 Table. In order to test whether these markers were specific to *A*. *cristatum* 6P chromosome, they were used to amplify target fragments using the genomic DNA of *A*. *cristatum*, Fukuho, and different types of disomic addition lines as templates. All the 15 SLAF-seq markers were present in *A*. *cristatum* and 5113 but absent in Fukuho and other addition lines (Fig 5), suggesting that these markers were *A*. *cristatum* 6P chromosome-specific. In order to further test whether these SLAF-seq markers could trace the alien *A*. *cristatum* 6P chromosomal segments instead of GISH, 15 SLAF-seq markers were used to genotype multiple genetic populations derived from 5113 and Fukuho (F_1_, BC_1_F_1_ and BC_1_F_2_ populations, with Fukuho as the recurrent parent), followed by GISH analysis. All the progenies positive for the 15 SLAF-seq markers showed GISH signals, and all the progenies with GISH signals were positive for the 15 SLAF-seq markers (Fig 6). These results suggested that the 15 SLAF-seq markers could be applied to trace 6P chromosomal segments instead of GISH.

### Chromosomal localization of the desirable genes using genetic populations

In order to explore the source of the genes conferring the desirable traits in 5113, genotypic and phenotypic data were analyzed in F_1_, BC_1_F_1_ and BC_1_F_2_ populations. As shown in Fig 3 and S1 Table, progenies of three populations could be categorized into two types based on the genotypic data: progenies with SLAF-seq markers and progenies without SLAF-seq markers. The progenies positive for SLAF-seq markers displayed obvious differences from those negative for SLAF-seq markers in nine yield-related traits, including the ratio between uppermost internode length and plant height ratio, flag leaf length, flag leaf width, spike length, grain number per spike, spikelet number per spike, kernel number in the middle spikelet, fertile tiller number per plant and whole growth period. All the progenies with SLAF-seq markers and 5113 exhibited similar phenotypes in nine traits, while all the progenies negative for SLAF-seq markers and Fukuho exhibited similar phenotypes. All these results suggested that genes conferring these traits were located on the *A*. *cristatum* 6P chromosomes of 5113. Besides, genes conferring powdery mildew and leaf rust resistance were also proved to be located on the *A*. *cristatum* 6P chromosomes of 5113. However, there were no significant differences in two traits (plant height and thousand-grain weight) between plants positive and negative for SLAF-seq markers, suggesting that genes conferring these two traits were not located on *A*. *cristatum* 6P chromosomes of 5113.

## Discussion

### The alien chromosome-specific markers could be effectively developed by the SLAF-seq technology

After introduction of alien chromosomes into the wheat genome, cytological methods can be used to detect the alien chromosomal fragments. However, utilization of these methods has been limited by the fact that they are time-consuming and labor-intensive. Recently, SLAF-seq markers have been successfully applied in detecting alien chromosomal fragments, due to their high throughput, high accuracy and low-cost [23]. In this study, 15 SLAF-seq markers specific to *A*. *cristatum* 6P chromosome were developed by the SLAF-seq technology. In the 5113 × Fukuho genetic populations, the presence/absence of SLAF-seq markers were consistent with the presence/absence of GISH signals, indicating that SLAF-seq markers can be used to detect the existence of *A*. *cristatum* 6P chromosome instead of GISH. In our further studies, these SLAF-seq markers will be used to trace *A*. *cristatum* 6P chromosomal segments in the translocation and introgression lines induced from 5113.

### 5113 was a novel wheat-*A*. *cristatum* disomic 6P addition line different from 4844–12

Six wheat-*A*. *cristatum* disomic 6P addition lines have been acquired in our laboratory, including 5113, 4844–12, 5114, 5106, II-26, and II-29-2i [31]. Although all the added pairs of *A*. *cristatum* chromosomes in these addition lines were homoeologous with the homoeologous group 6 of wheat, their genetic constitutions exhibited varying degrees of differences. In this study, differences between 5113 and 4844–12 were revealed by both genotyping and phenotyping. Compared with Fukuho, both 5113 and 4844–12 showed higher grain number, and resistance to powdery mildew and leaf rust. However, 5113 showed higher ratio between uppermost internode and plant height, larger flag leaf, longer spike length, more spikelet number per spike, more fertile tiller number, lower thousand-grain weight and longer whole growth period, compared with 4844–12. These differences between 4844–12 and 5113 were mainly attributed to the genetic rearrangements of *A*. *cristatum* 6P chromosomes. Similar phenomena were also reported in other species, such as *Leymus racemosus* [42], *Thinopyrum intermedium* [43], and rye [44]. Therefore, 5113 was a novel wheat-*A*. *cristatum* disomic 6P addition line different from 4844–12, which could be used as a starting material to produce novel wheat germplasms (translocation and introgression lines).

### 5113 could be used to transfer high-yield and disease resistance genes from *A*. *cristatum* into common wheat

Wheat-alien disomic addition lines are usually used as the starting material to transfer desirable agronomic traits into common wheat through chromosome engineering [45–48]. In this study, genes conferring multiple elite agronomic traits were located on the *A*. *cristatum* 6P chromosome of 5113 by using three different genetic populations (F_1_, BC_1_F_1_ and BC_1_F_2_ populations). In order to transfer these desirable genes from *A*. *cristatum* into common wheat, 5113 will be induced by ^60^Co-γ rays or homoeologous pairing induction. Translocation lines with small 6P chromosomal fragments are preferred to transfer elite genes into wheat. There were several examples in which translocation lines were successfully induced from their corresponding addition lines. For example, wheat-*Haynaldia villosa* translocation line T4VS⋅4DL induced from the disomic chromosome addition line DA4V were reported to display high resistance to wheat spindle streak mosaic virus [49]; wheat-*Haynaldia villosa* translocation line T2VS⋅2DL induced from the *T*. *durum*-*D*. *villosum* amphiploid was found with longer spikes and more kernels [50]; wheat-*Thinopyrum bessarabicum* translocation line T2JS-2BS⋅2BL induced from the CS-*Th*. *bessarabicum* alien telosomic addition line TJ04 displayed more fertile spikes per plant, longer spikes, more grains per spike and higher yield per plant [51]. Successful applications of translocation lines mentioned above were based on the knowledge of their genetic constitutions and agronomic traits. Therefore, chromosomal localization of these desirable genes is a fundamental prerequisite for the utilization of 5113 to enrich the germplasm resources for wheat breeding.

## Supporting Information

S1 Table Evaluation of the agronomic traits of 5113, 4844–12, Fukuho and genetic pupolations derived from 5113 and Fukuho. (XLS)

S2 Table The primer sequences of 15 SLAF-seq markers specific for *A*. *cristatum* 6P chromosome. (XLS)

## Abbreviations

Fukuho: Fukuhokomugi
GISH: Genomic in situ hybridization
LGs: Linkage groups
MI: Metaphase
PMCs: Pollen mother cells
SLAF-seq: Specific-locus amplified fragment sequencing
